# Impact of Source and Manufacturing of Collagen Matrices on Fibroblast Cell Growth and Platelet Aggregation

**DOI:** 10.3390/ma10091086

**Published:** 2017-09-15

**Authors:** Stefanie Böhm, Christine Strauß, Stefan Stoiber, Cornelia Kasper, Verena Charwat

**Affiliations:** 1 Technical Chemistry, Leibniz University Hannover, 30167 Hannover, Germany; stefanie.boehm@clariant.com (S.B.); cornelia.kasper@boku.ac.at (C.K.); 2 Department of Biotechnology, University of Natural Resources and Life Sciences, 1190 Vienna, Austria; christine.strauss@students.boku.ac.at (C.S.); stef.stoiber@chello.at (S.S.)

**Keywords:** collagen scaffolds, fibroblasts, platelet aggregation, 3D cell culture

## Abstract

Collagen is a main component of the extracellular matrix. It is often used in medical applications to support tissue regeneration, hemostasis, or wound healing. Due to different sources of collagen, the properties and performance of available products can vary significantly. In this in vitro study, a comparison of seven different collagen matrices derived from bovine, equine, and porcine sources was performed. As performance indicators, the scaffold function for fibroblasts and platelet aggregation were used. We found strong variation in platelet aggregation and fibroblast growth on the different collagen materials. The observed variations could not be attributed to species differences alone, but were highly dependent on differences in the manufacturing process.

## 1. Introduction

The extracellular matrix (ECM) component collagen is one of the most abundant proteins in the human body. It plays a pivotal role in providing tensile tissue strength and tissue repair due to its unique physical and biological properties including hemostatic function, biodegradability, enhancement of new collagen deposition, cell recruitment, control of proteolytic activity in chronic wounds, and low-antigenic response [1,2]. Based on its characteristics, collagen is one of the most widely used biomaterials in the field of tissue engineering, advanced wound care, and regenerative medicine, and is extensively investigated for clinical applications [3,4]. Collagen is a natural biopolymer, which can be isolated from different species and tissue sources as well as subjected to a variety of scaffolding procedures. Depending on the manufacturing approach, it is processed into fibers, meshes, porous sponges, or hydrogels [2]. 

The main sources for collagen are donated human tissue or large mammals such as cattle, horses, pigs, or sheep. Other sources, e.g., fish and jelly fish, are used as well, although less frequently [5,6]. Collagen is mainly derived from tissues such as skin, tendons, pericardium, or inner organs such as small intestine submucosa, forestomach, or bladder. As collagen is an evolutionary, highly conserved molecule, mammalian-derived collagen is generally well tolerated in human. During the healing process, collagen is enzymatically degraded and remodeled over time, and eventually replaced by the body’s own collagen [7,8]. 

To modify the properties of collagen, it can be blended with other ingredients such as polysaccharides e.g., alginate, oxidized regenerated cellulose (ORC), glycosaminoglycans, proteins e.g., elastin, and drugs such as antimicrobials e.g., silver, antibiotics, or enzymes e.g., thrombin [9,10,11]. This can lead to distinct changes in clinical outcome. It was shown that a combination of collagen and elastin performed best with regard to scar contraction in a deep dermal wound model in pigs [9,12].

Medical collagen products are often used as a mechanical scaffold to support cell invasion or as a sponge-like device that is able to store large amounts of fluids such as wound exudate or blood [13]. Moreover, its porous structure enables nutrient and oxygen supply throughout the matrix, thereby supporting wound healing and tissue regeneration. It provides a microstructure that is highly amenable for cell attachment, migration, and spreading [14,15]. This is not only triggered by the collagen lattice geometry, but even more by specific binding sites to which cells can attach either directly or through intermediary proteins [16]. It was shown that collagen is able to interact with cellular integrins and supports different cellular functions and regeneration via chemotactic signals [17]. 

An additional characteristic of collagen molecules is the activation of platelets. When platelets come in contact with collagen, a reaction cascade is started, which finally leads to an aggregation of platelets. The aggregated platelets form a clot that effectively reduces bleeding [18]. Importantly, the complete platelet aggregation cascade can only be induced by native collagen. Monomeric and fibrillar collagens effectively support platelet adhesion, whereas the native, triple-helical structure of collagen and the polymerization of the monomeric collagen is required for collagen-induced platelet aggregation and secretion [19,20,21]. A fast integrin mediated adhesion was shown for native but not for denatured collagen [22]. This illustrates the importance of maintaining the nativeness and active binding sites of collagen and thereby the quality of the collagen product during the manufacturing process.

**Figure 1:**
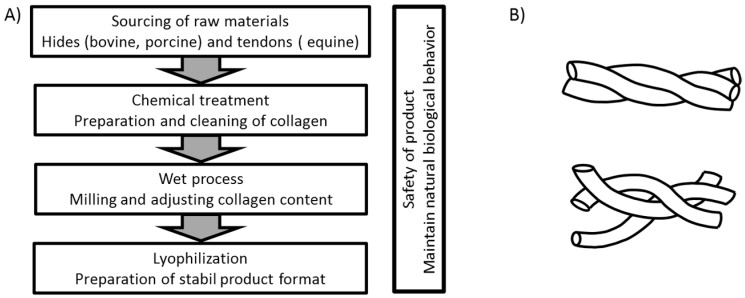
Collagen scaffolding. (**A**) Basic workflow for the manufacturing of collagen matrices compared in this investigation; (**B**) Top: Intact, native collagen characterized by an intact triple helix; Bottom: denatured collagen, triple helical structure destroyed.

In the production of collagen-based products, different methods can be used depending on the origin of the raw material and the intended application of the final product. A general overview of the process flow for the products used in this investigation is shown in Figure 1A. In each case, a thorough cleaning of the raw material is needed to eliminate potential biological hazards. For animal-derived products, the process has to ensure cell removal to avoid any inflammatory and rejection reactions based on remaining α-gal (galactose-alpha-1,3-galactose) epitopes of the animal source [23]. During the first step of production the materials are cleaned mechanically from other tissues and then subjected to chemical treatment to decellularize them [24,25]. This is often performed by a treatment of alkaline and or acid solutions for several hours or even days. In order to reduce the density of the source tissues the materials can be milled in consecutive steps and diluted with aqueous solutions [10,26,27,28,29]. Most manufacturing processes include lyophilization in order to achieve good shelf life at room temperature. By this sequence of process steps, an open porous structure is created that provides a large surface for cell interaction and the absorbance of fluids. Although this general procedure is used for the production of all materials included in this study, it is expected that even rather small differences in the production may result in a different performance of the final products. The use of chemicals and treatment conditions such as organic chemicals for degreasing or elevated temperature will influence the intactness of the collagen fibrils and may influence the effectiveness of the product. Thermal treatment of the collagen will lead to a destruction of the triple helix, resulting in an uncoiled, gelatin-like structure (see Figure 1B) that does not achieve native interaction with cell receptors. As each company uses their own variation of the general treatment regime, differences in the quality and functionality of the final products can be expected. 

The aim of this investigation was to show the impact of different collagen sources and influences that may derive from variations in the production process on the performance of the products in an in vitro approach. In order to analyze a more homogenous group of collagen matrices, we have focused on matrices made from tissue sources rich in collagen type I such as hides and tendon. The analysis of collagen matrices based on type II collagens found in cartilage, for example, is beyond the scope of this manuscript. Therefore, commercial available products such as Promogran, Suprasorb C, Nobakoll, and Kollagen resorb were compared to bovine, porcine, and equine collagen matrices manufactured based on the proprietary 2C Technology by MedSkin Solutions Dr. Suwelack AG. The according 2C-technology serves as a platform for customized wound care solutions and hemostat products. To estimate the functionality of collagen matrices for medical applications, the use as hemostat and scaffold for regenerative as well as for wound healing purposes was chosen. As fibroblasts and platelets are both able to interact biologically with native collagen via receptors, the aggregation of platelets and the growth of fibroblasts were selected as model systems.

## 2. Results

### 2.1. Matrix Evaluation in 3D Cell Culture

Cell-matrix interaction is a crucial factor for the clinical outcome of biomaterials applied as wound dressings and regenerative implants. In an in vitro setup we modeled the cell matrix contact and monitored cell spreading and activity.

**Figure 2:**
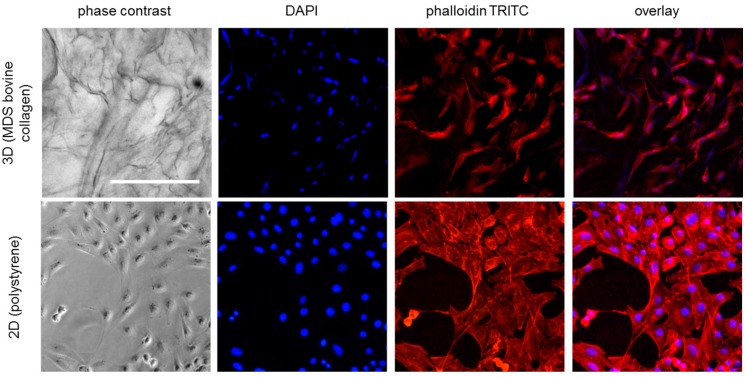
Microscopic images of NIH 3T3 fibroblasts in standard 2D culture on polystyrene surface and in 3D culture on MDS bovine collagen matrix (48 h incubation; 35,000 cells/cm² seeding density). Cells were stained with DAPI (blue, nuclei) and TRITC-conjugated phalloidin (red, f-actin). Scale bar represents 250 µm.

**Figure 3:**
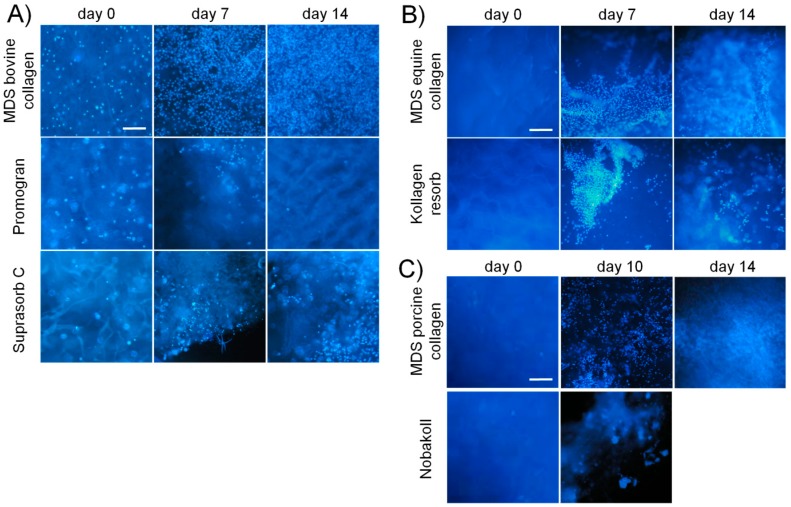
DAPI images of collagen matrices seeded with NIH 3T3 fibroblasts and incubated up to two weeks: (**A**) bovine matrices; (**B**) equine matrices; and (**C**) porcine matrices. Scale bars represent 200 µm.

**Figure 4:**
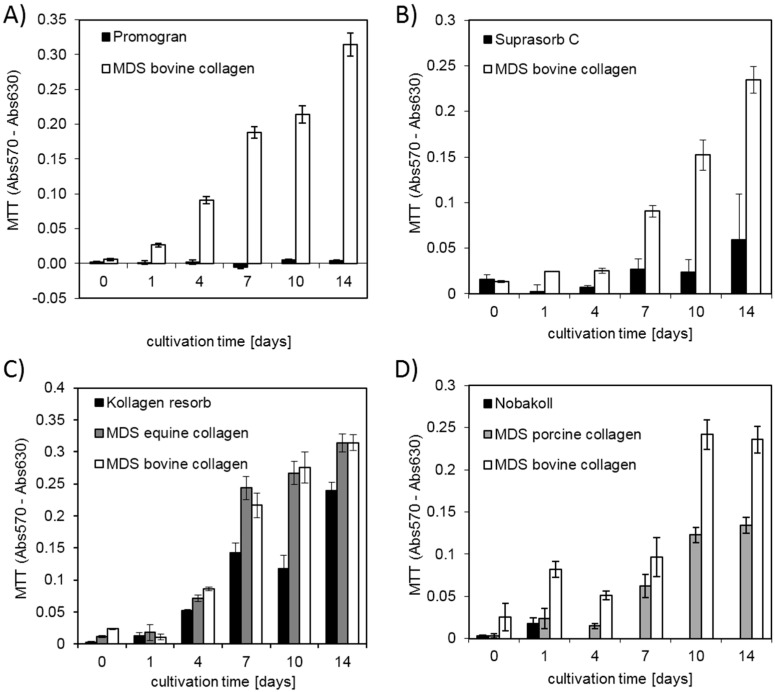
MTT absorbance values (absorption at reference wavelength and blanks subtracted) for up to two weeks of cell culture on different collagen matrix materials. (**A**,**B**) Bar charts for bovine collagen scaffolds of 15 mm and 6 mm diameters, respectively. Data of equine and porcine materials is shown in (**C**,**D**), respectively. Both (**C**,**D**) contain additional measurements of MDS bovine collagen for comparison between the different tests.

First, a qualitative characterization of cell attachment and distribution on the different matrices was performed. Fluorescence staining was employed to identify cells in the 3D matrix, since they cannot be distinguished from the matrix background in light microscopy (see Figure 2). DAPI staining was used to stain the cell nuclei while TRITC-phalloidin was applied for cytoskeletal staining. Phalloidin staining (Figure 2) revealed the cytoskeletal structure in the 2D samples and indicated cell shape in the 3D samples. However, DAPI staining provided less background and allowed better evaluation of cell distribution and number in the 3D samples, especially for high cell densities (see also Figure S1). DAPI pictures at reduced magnification were taken from the top side of each matrix after different incubation times (Figure 3). On all tested materials, bright blue spots representing the cell nuclei were found, confirming cell attachment. Generally, a relatively homogeneous distribution of cells on the collagen disks was observed, indicating reliable seeding procedures. Over time, the blue fluorescence signal increased for all matrix materials except Promogran and Nobakoll. Increasing numbers of nuclei further confirmed successful cell attachment to the substrate as well as viability and proliferation. After two weeks of cultivation, most materials were completely crowded with a densely packed cell layer. However, on Promogran disks cell numbers appeared similar on day 7 as directly after seeding, indicating no cell proliferation. On day 14, only a few nuclei could be identified on the matrix, which suggested cell detachment or cell death. For Nobakoll, strong matrix degradation was observed starting at day 2. On day 10, only a few remnants of the material with low cell numbers were present in the well. On day 14, the matrix had been completely degraded, therefore no picture could be taken. Similarly, the Suprasorb C disks also showed strong degradation over the experimental time. As expected for 3D cell cultures, relatively high background fluorescence was observed in all samples as a result of cells growing not only in a single layer on the matrix surface but spreading deeper into the scaffold. Due to cell distribution in the Z-direction and background fluorescence, no quantification of the fluorescence pictures regarding fluorescence intensity or count of nuclei was performed. DAPI staining provided useful information; however, further experiments were performed to obtain quantitative measurements on cellular activity by using the MTT assay and CellTiter-Blue assay. In the assay, absorption values are proportional to the overall metabolic activity in the sample, which is influenced by cell number, viability, proliferation, and metabolic activity per cell. MTT results are plotted in Figure 4. MedSkin Solutions Dr. Suwelack AG (MDS) bovine collagen was always added as a control to allow comparison between the assays. For most matrix materials MTT signals rose over the incubation time, indicating increasing numbers of viable cells. The strongest cell proliferation was observed on MDS bovine and equine collagen. Slightly lower cell growth was found on Kollagen resorb (significant from day 7 on), followed by MDS porcine collagen (significantly lower than MDS bovine collagen on days 1, 10, and 14). The weak signal increase in Suprasorb C samples can be attributed to the considerable degradation of this material over the cultivation time. MTT signals of Promogran stayed very low over the entire cultivation period, confirming that cells did not proliferate on this material. During cell cultivation, it was noted that the medium color in wells containing Promogran rapidly changed from red to yellow, indicating a pH-shift towards acidic conditions. A low pH of 6.5 was confirmed by pH measurements, which is clearly below the ideal range of 7.2–7.4 for complete cell culture media. For Nobakoll, MTT results could only be obtained at day 0 and day 1 due to strong matrix disintegration. Overall, the metabolic activity results confirmed the initial observations based on nuclear staining. 

**Figure 5:**
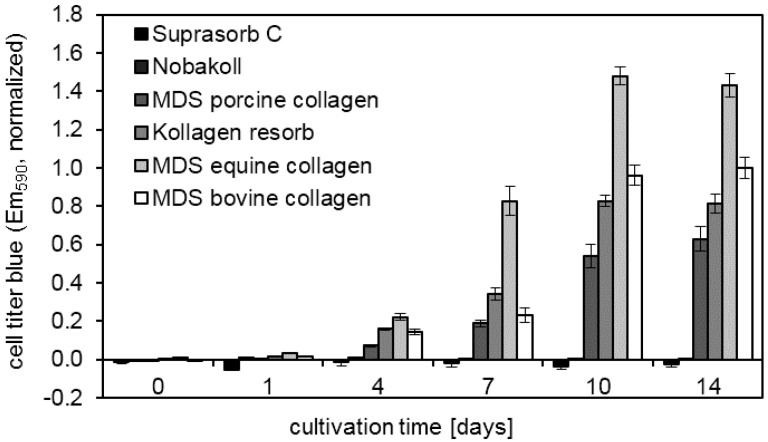
CellTiter-Blue assay indicates changes in metabolic activity up to two weeks after NIH 3T3 fibroblast seeding on different types of collagen disks.

In an attempt to obtain metabolic information from the Nobakoll samples, an additional test was performed. The CellTiter-Blue assay works similarly to the MTT assay based on the intracellular conversion of a dye. However, the CellTiter-Blue protocol is simpler and requires only a single pipetting step. It was possible to acquire measurement results for all timepoints, despite the loss of solid matrix (see Figure 5). The constantly low read out for Suprasorb C and Nobakoll can be attributed to matrix degradation and the resulting loss in the cell adhesion area. For the other matrix materials, results comparable to the MTT assay were found. Only for MDS equine collagen were surprisingly high values recorded from day 7 to 14. Statistical analysis revealed significant differences (*p* < 0.001) between all pairs of materials except Suprasorb C vs. Nobakoll on days 10 and 14 and between all except Suprasorb C vs. Nobakoll and MDS porcine collagen vs. MDS bovine collagen on day 7. On day 4, MDS equine collagen results were significantly higher than all others (*p* < 0.05), while Suprasorb C and Nobakoll values were significantly lower than all others except Nobakoll vs. MDS porcine collagen. On day 1, only Suprasorb C vs. MDS equine collagen showed a significant difference (*p* = 0.088). On day 0, no significant differences were found. CellTiter-Blue assay was not applied to Promogran samples, since the assay read out could be altered by the reduced pH values. By contrast, in the MTT assay a solution containing HCl is added to dissolve the crystals, resulting in acidic pH regardless of the original sample pH. 

### 2.2. Platelet Aggregation Test 

**Figure 6:**
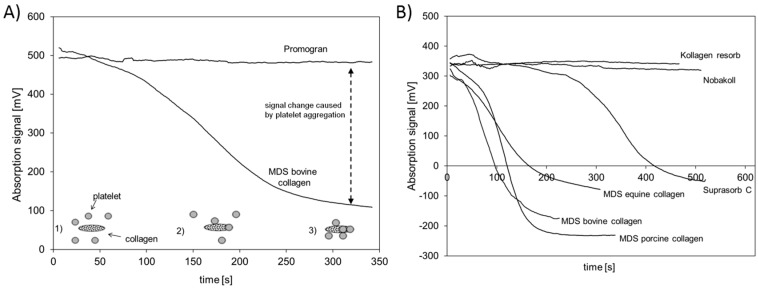
Platelet aggregation assay: (**A**) shows the concept of the platelet aggregation assay using Promogran and MDS bovine collagen as example materials. Platelets (grey circles) in plasma cause an absorption of light (1). They adhere (2) and aggregate on collagen (spotted ovals) (3) and lead to a clearance of the plasma. The signal change before and after the aggregation is used as a measure for the effectiveness of the collagen to trigger platelet aggregation; (**B**) Shows time-dependent changes in the aggregation status for MDS bovine, equine, and porcine collagen, as well as Suprasorb C, Nobakoll, and Kollagen resorb.

In this approach an optical platelet aggregation test [30] was used to estimate whether collagen-based wound dressings still possess native structure. It is assumed that a fast and reliable triggering of platelet aggregation is proof of the nativeness of collagen, whereas a failure or delay in aggregation indicates a loss in the native behavior of the collagen. The general principle of the test is shown in Figure 6A. In the beginning, platelets are homogenously distributed in the solution and cause a high absorption signal (1). Over time the platelets aggregate and stick to the collagen and are depleted in the fluid phase, which causes a decrease in the absorption signal (2, 3). Average absorption curves obtained during platelet rich plasma (PRP) incubation with the different collagen scaffolds are depicted in Figure 6. To estimate the speed of aggregation, the inflection point of the aggregation curves was calculated (see Table 1) and an early time for the inflection point is regarded as a fast triggering of the platelet aggregation. 

Two groups of collagen materials could be identified. The first group did not affect the aggregation at all and did not cause any signal change over time. Promogran, Kollagen resorb, and Nobakoll belong to this group. The test was tracked for at least 300 s and during this time the materials did not induce any aggregation. The second group of materials resulted in a signal decrease, indicating an aggregation of platelets. Samples made of MDS bovine, porcine, and equine collagen were able to induce clotting rapidly. Suprasorb C was able to induce platelet aggregation but at a later timepoint than the other materials of this group. As a control, PRP without the addition of collagen was tested and no induction of aggregation was observed (data not shown). The time of the inflection points (Table 1) confirmed these observations. MDS bovine, equine, and porcine collagen showed clearly shorter times than Suprasorb C. For all other materials, no calculation could be performed due to a missing aggregation. 

## 3. Discussion

The use of collagen for medical products has been established for more than four decades. Collagen scaffolds are used in hemostasis, wound healing, and regenerative medicine, e.g., as a dermal template or carrier for implantable scaffolds.

In order to provide an optimal environment for cellular ingrowth as well as platelet aggregation, the nativeness of the collagen as well as its helical structures and cellular binding sides need to be retained. Brown et al. conducted animal studies to characterize the influence of different ECM-based implants on the regeneration of surgical wounds. Materials that provided a near native composition showed the most promising results [31]. 

The nativeness of the products strongly depends on the manufacturing process. A thorough but mild decellularization, strict control of temperature, and an avoidance of residual chemicals, for example, is needed to obtain effective products in order to minimize or avoid encapsulation and inflammatory reactions [25]. All collagen sheets used in this study were manufactured by lyophilization; however, exact process parameters are unknown for the commercial products. Since different ways of extraction, processing, and sterilization may influence the biologic response with regard to cell attachment and migration as well as hemostasis, the collagen matrices were compared with reference materials of bovine, equine, and porcine origin fabricated at MedSkin Solutions Dr. Suwelack AG that were processed using essentially similar protocols. It is noteworthy in this regard that it was previously shown that the microstructure of collagen-based matrices such as density or fiber orientation influences cellular morphology and behavior [32,33]. Raster Electron Microscopic Analyses of the collagen matrices used in this study revealed structural differences of the materials that might further influence their performance with regard to fibroblast adhesion and growth (data not shown). Structural differences are not only a function of collagen composition, but also of processing parameters such as concentration or freezing rate [34,35]. 

In addition to the impact of the manufacturing process, this gives the opportunity to compare the influence of the collagen source on the matrix performance. The collagen sources used in this investigation were bovine and porcine hides as well as equine Achilles tendons. In this regard, Angele et al. compared the use of bovine and equine tendon collagen, which were basically prepared in the same way for use in cell culture. They found a higher thermal stability of equine collagen compared to bovine collagen and a tendency of equine collagen to rupture under higher mechanical resistance compared to bovine collagen matrices [36].

All materials investigated in this study are intended to be used as wound dressings or hemostats; therefore, fibroblasts and platelets were chosen as clinically relevant cell types to analyze the influence of the manufacturing process as well as the collagen source on the performance of the materials [37,38].

One of the main functions of collagen in wounds is the guidance of cell growth and migration during the proliferation and remodeling phase of the healing process [5,39]. These favorable properties for cell attachment and growth make collagen a promising material for clinical application. Our study confirmed that successful cell attachment was possible on the different collagen-based matrix materials. DAPI staining showed that cell attachment was clearly visible on all bovine matrices already on day 0. However, initial pictures of porcine and equine materials showed only a very sparse presence of cells. This observation suggests that cell attachment occurred at different speeds on the different materials. Cells might have not yet been completely attached to some of the materials and were washed away during the staining procedure. Another possibility is that cells were absorbed deeper into the porcine and equine scaffolds due to different wettability and pore size. However, DAPI pictures and metabolic activity analysis at days 7 and 14 showed cell survival and proliferation. This finding confirmed successful cell seeding, even on materials that initially showed a low presence of cells. Qualitative findings obtained from DAPI staining were confirmed by using the quantitative MTT and CellTiter-Blue^®^ metabolic assays. Overall MDS bovine and equine collagen showed highest cell proliferation, followed by Kollagen resorb and MDS porcine collagen. Low metabolic activity was detected for Suprasorb C and Nobakoll due to material degradation and for Promogran due to pH reduction that interfered with cell growth. This effect can be attributed to the ORC fraction of the product Promogran. A drop in pH level was described for oxidized cellulose hemostatic agents, although some authors describe differences between investigated media types [40,41]. The shift to a more acidic value might on the one hand occur by degradation, as the depolymerization leads to the release of acidic groups [42]. On the other hand, the fibroblasts themselves might lower the pH. Wagenhäuser et al. hypothesized that the fibrous components of the ORC material might alter cell function, leading fibroblasts to release acid or to undergo apoptosis.

Another important parameter for biomaterials in clinical application is the balance between stability and degradability. Stability is needed to provide a scaffold for cell growth and migration as well as protection from matrix metalloproteinases (MMPs). In chronic wounds, elevated levels of MMPs cause excessive collagen degradation. Here, collagen-based wound dressings can help to re-establish an equilibrium since they act as an additional substrate for MMPs and thereby protect the patient’s collagen from breakdown. In this regard, a recent study by Tati et al. [43] (submitted) showed a higher capacity of wound dressings that contain native collagen to bind and inactivate MMPs. Nevertheless, all tested materials are bioresorbable and designed to eventually degrade and be replaced by freshly grown patient tissue. Degradation products enhance the migration of fibroblasts, epithelial, and vascular endothelial cells into the wounded region to form the granulation tissue [44]. In our study, we found strong differences in the degree of degradation of the different materials. For most collagen matrices, no obvious loss in material amount, size, or structure was observed during our two-week experimentation time, indicating relatively slow degradation. However, Nobakoll and Suprasorb C started to degrade within a few days of cultivation time and very little to no material was left by the end of the experiment. Together with the matrix, the adhering cells were gradually lost, resulting in low metabolic activity signals in the MTT assay. 

Our in vitro findings are intended to give a first estimation of the behavior in a wound, but it has to be considered that in vivo a more complex set of different cell types, bodily fluids, possible microbial contamination, and mechanic agitation influences the matrix degradation process. 

In addition to the analysis of cell growth on the different collagen matrices, the aggregation of platelets on the matrix surface was investigated. Collagen is regarded as being beneficial in blood coagulation, as the collagen fibrils enable platelets to adhere on the surface and in this way enhance the natural aggregation and degranulation, initiating the formation of a physiological platelet plug [11,45]. Therefore, collagen matrices are widely used as hemostatic dressings. Note that the aggregation cascade can only be induced by native collagen as it provides the intact binding sites needed for interaction with the platelets [22]. All materials used in this study except Nobakoll and Suprasorb C claim to support the hemostasis process in wounds. Although it can be assumed that all materials will contribute to hemostasis by absorbing diffusing blood and reducing bleeding based on their capillary activity, the ability to induce the aggregation of platelets varies to a large extent. In the present study, no aggregation was observed for Kollagen resorb, Nobakoll, or Promogran. Bovine, porcine, and equine collagen of MDS induced aggregation effectively, whereas aggregation using Subrasorb C was somewhat delayed. These findings suggest that the native structure in bovine, porcine, and equine collagen of MDS is mostly intact and available at the surface, resulting in a good platelet-collagen interaction. For Kollagen resorb, Nobakoll, and Promogran, this interaction was not observed, which might be due to a destruction of the native structure or a blocking of the surface due to product composition. Promogran is composed of a mixture of ORC and collagen (45%:55%), so the surface of the collagen might be compromised by ORC fibers. Suprasorb C reveals some potential to induce aggregation, but the delay suggests that less native collagen structures are accessible for platelet interaction. In this context, Wagner et al. used an in vitro approach by testing platelet activation in PRP and clotting time in whole blood samples using collagen, gelatin, and ORC matrices. They ranked the overall hemostatic activity as a combination of the relative performance of each test conducted, suggesting that collagen was the most effective, followed by gelatin and ORC [46]. Interestingly, in the current study different collagen products were used and they showed varying activity which might be based on different production processes. A similar suggestion was made by Eloy et al. Hemostats were tested in vitro for their ability to contribute to the plasmatic and cellular activation steps of the coagulation cascade. The potential of collagen to act as a powerful hemostatic agent was confirmed, but significant changes between different commercial collagen preparations were also observed. These differences may be due to different extraction and purification procedures, or different modes of sterilization of the products, resulting in changes in the bioactive conformational structure of the collagen and thus a limited extent of aggregation [47]. 

As most of the products used in this study are designed as wound dressings, the activation of platelets are not the foremost indication of these products. However, it should be kept in mind that natural hemostasis is the first step in wound healing [48]. The degranulation of platelets enables the release of platelet-derived growth factor (PDGF) that is beneficial in wound healing. Treatment of injuries or inflamed body parts with activated PRP showed good clinical results, indicating that the activation of platelets by collagen might be beneficial in the wound healing cascade, resulting in a structured regeneration of damaged tissue [4,49,50]. 

Taken together, our analysis shows that collagen matrices that originated from the same source but were processed differently (e.g., bovine hides) explore a more pronounced difference with regard to performance in cell growth and platelet aggregation compared to collagen matrices from different animal sources that were processed under similar conditions. We therefore conclude that although there is an influence of the collagen source on its properties, the processing of the collagen seems to have a stronger impact. A manufacturing process that maintains an open porous structure and nativeness of the collagen, and thereby maintains its binding sites for cell attachment, ensures optimal cell migration and proliferation.

## 4. Materials and Methods 

### 4.1. Cell Culture

NIH 3T3 cells (ACC 59, DMSZ, Braunschweig, Germany) were cultivated in Dulbecco’s modified Eagle’s medium (DMEM; D5648; Sigma Aldrich, Munich, BY, Germany) supplemented with 10% fetal calf serum (FCS; A15-101; PAA Cell Culture Company, Pasching, Austria) and 1% penicillin/streptomycin (P4333; Sigma Aldrich), in a humidified environment at 37 °C and 5% CO_2_ (standard incubator Hera Cell 240, Heraeus Holding GmbH, Hanau, Germany). Cell culture reagents and chemicals were obtained from Sigma Aldrich unless indicated differently. Culture flasks and well plates were purchased from Sarstedt AG (Nümbrecht, Germany). 

### 4.2. Collagen Matrices 

In this comparative study, seven different collagen matrix materials were investigated: Promogran, Suprasorb C, Nobakoll, and Kollagen resorb as well as sterile bovine, porcine, and equine collagen matrices provided by MedSkin Solutions Dr. Suwelack AG (MDS), Billerbeck, Germany (later referred to as MDS bovine collagen, MDS porcine collagen, and MDS equine collagen). The MDS collagen matrices were manufactured by the same method, but using different animal origins. In brief, bovine and porcine collagen was made of hides. The collagen-rich dermal layer was used for further processing. The dermal layer was treated under alkaline conditions to remove residues and then adjusted to an acidic pH value. The skin was then milled and diluted to obtain the final collagen concentration prior to freeze-drying of the material. Matrices with a final thickness of 2 mm were sterilized using gamma radiation. Equine collagen was produced in a similar way. Equine tendons were thoroughly defleshed before starting the alkaline treatment. The MDS collagen preparations are intended to be used for tissue regeneration, as wound dressing materials as well as for hemostatic purposes.

Promogran (REF: M772123) is made of 55% bovine collagen and 45% ORC and was purchased from Systagenix Wound Management GmbH, Hamburg, Germany (parent company: Acelity, San Antonio, TX, USA). Promogran is prepared as a sterile, 3-mm thick porous sheet by lyophilization of a collagen and ORC fiber suspension. Its main clinical use is topical application in chronic non-healing wounds, where it forms a gel and absorbs exudate during a slow degradation phase. Additionally, Promogran can be used on bleeding surface wounds. 

Suprasorb C (REF: 20,483, Lohmann & Rauscher GmbH & Co. KG, Neuwied, Germany) is an 8-mm thick collagen matrix prepared from bovine hides (Corium) by freeze-drying. Its main application is treatment of skin areas with large superficial tissue defects, especially chronic stagnant wounds. The porous structure of Suprasorb C provides a capillary structure for liquid absorption to remove wound exudate. The collagen matrix is intended to support skin cell ingrowth. The product is manufactured aseptically. 

Kollagen resorb (REF: RK1209, Resorba Medical GmbH, Nürnberg, Germany) is a sterile 3–4-mm thick, soft, dimensionally stable collagen sponge for local hemostasis. It is made of collagen fibrils of equine origin (horse Achilles tendon) and used as a hemostatic agent in cases of venous, capillary, and diffuse bleeding as well as a temporary skin cover. 

Nobakoll (REF: 781,110, NOBA Verbandmittel Danz GmbH, Wetter, Germany, manufactured by MBP GmbH, Neustadt-Glewe, Germany) is a 5-mm thick, sterile collagen sponge made of porcine hide (corium) that is employed for chronic wounds and wound healing by secondary intervention.

### 4.3. Matrix Preparation and Cell Seeding

All matrix materials were provided as sheets with thicknesses of a few millimeters. Tests were performed in 24- and 96-well microtiter plates. For in vitro testing, circular disks with diameters of 6 mm were prepared using a hollow punch of the appropriate size. For Promogran, a larger format (15-mm disks) with a lower volume per cell was selected due to undetectably low MTT signals. As a reference, MDS bovine collagen was analyzed in both formats. The matrix pieces were transferred to a 24-well plate (15-mm disks) or 96-well plate (6-mm disks). Before cell seeding, the materials were sterilized for 3 h by UV light treatment inside the laminar flow workbench. No alterations of the materials were observed after UV treatment (neither macroscopically, nor by light microscopy, nor electron microscopy; data not shown). All subsequent cell cultivation experiments were carried out under sterile standard conditions. The matrices were moistened with cell culture medium (100 µL for 15-mm disks and 20 µL for 6-mm disks) and incubated for a few minutes. Then, cell suspension was pipetted on top of each matrix to inoculate with 100 cells/mm^2^ (100 µL containing 18,000 cells for 15-mm disks and 60 µL containing 3000 cells for 6-mm disks, unless indicated differently) and incubated for 1.5 h to allow cell adhesion. Next, 200 or 500 µL cell culture medium were added to the 6- and 15-mm disks, respectively. A medium exchange was performed on days 7, 10, and 12.

### 4.4. Nuclear Staining 

To visualize the cell distribution and number, 4′,6 diamidino-2-phenylindole dihydrochloride (DAPI; D8417; Sigma Aldrich) staining was performed. The blue fluorescent nuclear dye preferentially binds to dsDNA and less effectively to RNA, resulting in bright nuclear staining of mammalian cells. The matrices were washed with phosphate buffered saline (PBS; P38135; Sigma Aldrich) and then the cells were fixated on the matrices with ice-cold ethanol (96%) for 1 h at 4 °C in the dark. After rinsing the matrices with PBS, the DAPI solution was added (1:500 DAPI–stock solution (1 mg/mL) in PBS) and incubated for 30 min, at 37 °C in the dark. Subsequently, cells were visualized using a fluorescence microscope (Olympus IX 50 Olympus Corporation, Tokyo, Japan) equipped with a DAPI filter block.

### 4.5. F-Actin Staining 

For the staining of cytoskeletal f-actin fibers, TRITC-conjugated phalloidin (R415; Molecular Probes; reconstituted to 200 units/mL in methanol) was used. The cells were fixated with 4% paraformaldehyde (F8775; Sigma Aldrich) in PBS for 20 min at ambient temperature on an orbital shaker. Then, 0.1% Triton-X 100 in PBS was applied for 2 min for membrane permeabilization. The samples were rinsed with PBS, and submerged in a staining solution containing 1% FCS, 4 units/mL phalloidin, and 2 µg/mL DAPI in PBS. After 30 min incubation at ambient temperature on an orbital shaker, the samples were rinsed twice with PBS and analyzed using a fluorescence microscope.

### 4.6. Metabolic Activity Assay 

Viability of the cells was analyzed by (3-(4,5-dimethylthiazol-2-yl)-2,5-diphenyltetrazolium-bromid) (MTT) assay (M5655, Sigma Aldrich) on days 0 (directly after seeding), 1, 4, 7, 10, and 14. The MTT assay is a widely used spectrophotometric method for viability evaluation through the metabolic activity of mitochondria in living cells. For the analysis, the matrices were transferred to a fresh plate. Materials of 15-mm diameters were incubated with 660 µL MTT solution (600 µL DMEM + 60 µL MTT stock solution (5 mg/mL PBS, sterile)) for 4 h at 37 °C and 5% CO_2_. Thereafter, 600 µL solubilization solution containing 10% SDS (*w*/*v*) in 0.01 M HCl was added and incubated overnight at 37 °C. The next day, the dissolved solution was transferred to a 96-well plate (100 µL/well) for testing. The absorption was measured at 570 nm (and 630 nm as a reference value) in a microplate reader (Model 680, Multiskan Thermo Fisher Scientific Inc., Waltham, MA, USA). Cell culture medium on matrix materials without cells was used to generate blank values. For the experiments with 6-mm diameter disks, the applied volumes were 110 µL MTT solution and 100 µL solubilization solution. Replicate numbers were four for the 15-mm disks and six for the 6-mm disks. Additionally, a CellTiter-Blue assay (G8080; Promega, Mannheim, Germany) was performed. In this fluorometric viability assay, the metabolic activity of the cells is measured by the conversion of the indicator dye resazurin. The matrices were transferred to a fresh plate and 100 µL cell culture medium and 20 µL CellTiter-Blue were added to each well. After 2.5 h, 100 µL per well were transferred to a fresh well and emission was measured at 590 nm with 544 nm excitation (Fluoroskan Ascent Thermo Fisher Scientific Inc., Waltham, MA, USA). All data was normalized to MDS bovine collagen at day 14. Two-way ANOVA (with Tukey multiple comparisons test) was performed for statistical analysis.

### 4.7. Platelet Aggregation 

The materials were tested in an platelet aggregation assay according to Born [30]. This test is based on an optical change of turbidity PRP. When platelets are activated in PRP and start to aggregate, a clearance of the solution can be observed. Speed and magnitude of the reduction of optical density are parameters for effective aggregation. The photometer used for aggregation was operated with an light-emitting diode (LED) light source (660 nm) to detect the absorption of the PRP. The cuvette was heated to 37 °C by using a flow through a water bath system. The change of absorbance was tracked by a voltmeter with an A/D converter (Voltcraft digital multimeter VC 840, Conrad Elektronik, Hirschau, Germany) and the data was transferred to a personal computer using the software RealView 1.0 (Abacom, Ganderkesee, Germany). 

For testing the aggregation, porcine blood was used. It was treated with sodium citrate (3.2%) to avoid clotting. The blood was centrifuged for 10 min at 200 × *g* (Centrifuge: Heraeus Biofuge Stratos, Thermo Fisher Scientific, Vienna, Austria) and the platelet-rich supernatant was collected (PRP). Then, 1.4 mL of PRP was transferred in a cuvette and a circular sample with a diameter of 8 mm was added. The cuvette was placed inside the photometer, which was adjusted to 37 °C. Under vigorous stirring, which is required to activate the aggregation, the change of optical density (wavelength: 660 nm) of the solution was tracked and recorded for at least 5 min and up to 10 min. The change in optical density was expressed in mV (output of the device). Tests were performed in triplicate for the bovine materials and in duplicate for all other samples. The general principle of the Born test is depicted in Figure 6A.

To quantify the aggregation, the inflection point of the clotting graph was calculated. To reduce background noise a moving average over 10 s was calculated, and based on these data the inflection point was calculated. The time until the inflection point was considered as a measure to estimate the speed of aggregation. 

## 5. Conclusions

The analysis of collagen harvested from bovine, porcine, and equine sources revealed that, in principle, all three species are suitable for cellular growth and platelet aggregation. Overall, bovine and equine collagen performed better than the porcine collagen matrix. However, the structural features such as the native collagen triple helical structure as well as the cellular binding sites influenced by the manufacturing process show a higher impact. The biologic response of cells and platelets to the different products varied widely in this regard. Although not all different production processes are known in detail, it can be concluded that the more efficiently the structural integrity and biological activity of the collagen can be maintained during manufacturing, the more physiological cellular interaction will be observed. 

## Figures and Tables

**Table 1:** Inflection points of platelet aggregation curves. (n.d.: not detectable).

Material	Time of Inflection Point (s)
MDS bovine collagen	67
MDS porcine collagen	113
MDS equine collagen	97
Suprasorb C	338
Kollagen resorb	n.d.
Nobakoll	n.d.
Promogran	n.d.

## Supplementary Materials

The following are available online at www.mdpi.com/1996-1944/10/9/1086/s1, Figure S1: Microscopic images of NIH 3T3 fibroblasts in 3D culture on MDS bovine collagen matrix.
