# Supplementary material for: Impact of Source and Manufacturing of Collagen Matrices on Fibroblast Cell Growth and Platelet Aggregation
Source: Materials (Basel). 2017 Sep 15;10(9):1086. doi: 10.3390/ma10091086 (PMC5615740; doi:10.3390/ma10091086)
Supplement: Supplementary file 1 [file materials-10-01086-s001.pdf]

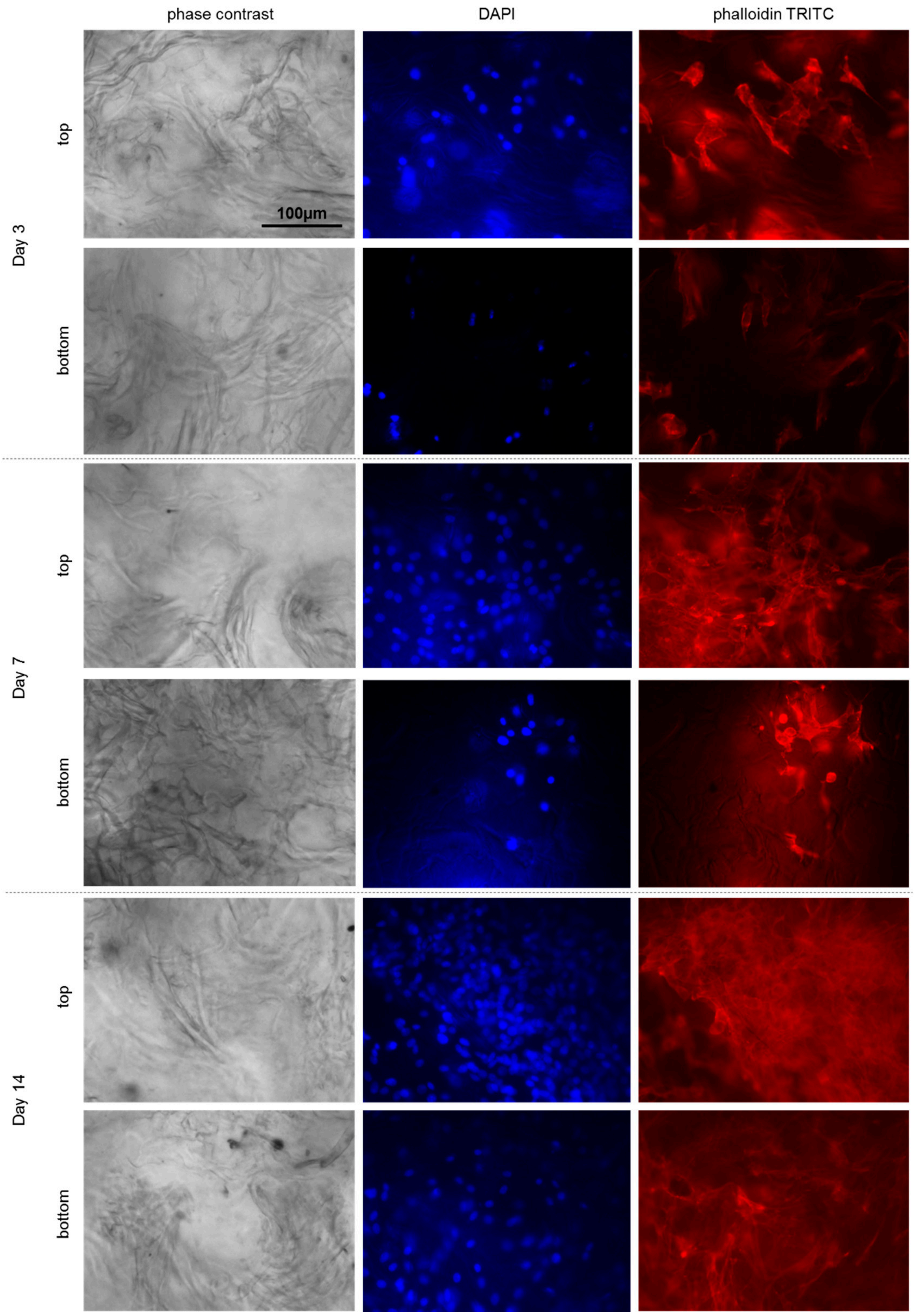

Figure S1. Microscopic images of NIH 3T3 fibroblasts in 3D culture on MDS bovine collagen matrix. Cells were were fixated and stained with DAPI (blue, nuclei) and TRITC-conjugated phalloidin (red, f-actin) after 3, 7 or 14 days incubation. Representative phase contrast and fluorescence images are shown for the top and bottom side of the matrix.
